# Trends in economic burden of type 2 diabetes in China: Based on longitudinal claim data

**DOI:** 10.3389/fpubh.2023.1062903

**Published:** 2023-04-18

**Authors:** Xinyi Liu, Luying Zhang, Wen Chen

**Affiliations:** School of Public Health, Fudan University, Shanghai, China

**Keywords:** diabetes, economic burden, diabetes-related cost, complications, China

## Abstract

**Objective:**

Diabetes is a major health issue in China that has a significant economic burden on society. Understanding the economic impact of diabetes can help policymakers make informed decisions about healthcare spending and priorities. This study aims to estimate the economic burden of patients with diabetes in an urban setting in China and to identify the impact of hospitalization and complications on health care costs for people with diabetes.

**Methods:**

The study was conducted in a sample city located in eastern China. All patients diagnosed with diabetes before January 2015 were identified from the official health management information system, and their social demographics and records of their health care uses and costs were extracted from the claim database from 2014 to 2019. Six groups of complications were identified according to ICD-10 codes. The diabetes-related direct medical cost (DM cost) was described for patients in stratified groups. A multiple linear regression model was applied to identify the effect of hospitalization and complications on the DM cost of diabetic patients.

**Results:**

Our research included 44,994 patients with diabetes, the average annual DM costs for diabetic patients increased from 1,292.72 USD in 2014 to 2,092.87 USD in 2019. The costs of diabetes are closely related to hospitalizations and the type and number of complications. The average annual DM cost of patients who were hospitalized was 2.23 times that of those without hospitalization, and it rose as the number of complications increased. Cardiovascular complications and nephropathic complications were the complications that had the greatest impact on patients’ DM costs, increasing by an average of 65 and 54%, respectively.

**Conclusion:**

The economic burden of diabetes in urban China has increased significantly. Hospitalization and the type and number of complications have significant impacts on the economic burden of patients with diabetes. Efforts should be made to prevent the development of long-term complications in the population with diabetes.

## 1. Introduction

Diabetes has become one of the most important public health issues worldwide in the 21st century and has been identified as one of five priority noncommunicable diseases by the World Health Organization in its Action Plan to address noncommunicable diseases challenges (1). According to the Diabetes Atlas (10^th^ edition) released by the International Diabetes Federation at the end of 2021, the number of adults with diabetes worldwide reached 537 million (10.5%), with an increase of 74 million compared with 2019, and global diabetes-related health expenditures were estimated to be at least $966 billion (2). With the rapid development of the economy and culture, the prevalence of diabetes in China has risen sharply. According to the Guidelines for the Prevention and Treatment of Type 2 Diabetes in China (2020 Edition), the overall prevalence of diabetes among adults has reached 11.2%, and the number of adults with diabetes is estimated at 141 million (3), with an undiagnosed ratio of 51.7% (2). The death toll from diabetes was nearly 1.4 million, and diabetes-related health expenditures reached 165.3 billion US dollars (USD) (3), which implies a considerable economic burden.

The health complications arising from diabetes posed obvious and rising health costs for people with diabetes, their families, and health care systems (3–5). Studies have consistently shown that patients with diabetes have an increased risk of kidney, eye, foot, cardiovascular and cerebrovascular complications, and peripheral nerve lesions, which can easily lead to high medical costs (6, 7). The cost of treating cardiovascular disease accounted for 35.3% of the total medical cost of patients with diabetes in the United Kingdom (8) and accounted for 27% of the total cost of treating diabetes in the United States (9). In addition, hospitalization caused by complications is also a factor that significantly affects the economic burden of patients with diabetes (10, 11). As planning and evaluating strategies to deal with the increasing diabetes burden requires accurate and comprehensive information on current diabetes-related costs and the economic burden of patients with diabetes (10, 11), it is crucial to continuously track and monitor the trend features of health care costs for people with diabetes based on real-world data.

Some studies have estimated the economic burden of diabetes in China, with most of them adopting a cross-sectional design and using data collected from several hospitals or household surveys (12–19). Therefore, there is still a research gap in this area in China. Firstly, there is a lack of recent and comprehensive studies that measured the economic burden of diabetes based on claim data, which contains individual health care utilization and cost information more complete and accurate than data from sampling hospitals or surveys. Secondly, previous studies failed to present the trend of the economic burden of diabetes over time. Thirdly, economic burden of diabetes in some studies were underestimated as costs of diabetes-related complications or related hospitalization were not included in calculation. Therefore, the aim of this study is to quantify the economic burden of diabetes in an eastern city in China from 2014 to 2019 using electronic claim data. This will provide a long-term picture of the economic burden of type 2 diabetes in China, which can help inform and motivate related strategies for diabetes control and prevention.

## 2. Methods

### 2.1. Setting

The study was conducted in a sample city (hereafter, H city) located in eastern China, which had 10.36 million long-term residents in 2019. The *per capita* Gross Domestic Product (GDP) was 22,102 USD, while the *per capita* disposable income of residents was 8,589 USD (20). As a provincial capital city in eastern China, H city is also one of the core cities along the eastern coast of China, and it has become a typical representative of cities in eastern China considering social and economic development. With a high degree of digital development, high-quality electronic health records in H city can be obtained from its well-developed health information system.

### 2.2. Data source

The patient population consisted of all patients diagnosed with diabetes before January 2015 in H city, covered by the Urban Employee Basic Medical Insurance (UEBMI) and Urban and Rural Residents Basic Medical Insurance (URRBMI), the two basic medical insurances. We included patients who had at least one explicit outpatient visit record per calendar year from 2014 to 2019. Data were extracted from two separate databases of H city in the years 2014–2019: (1) the official health management information system, and (2) the medical insurance claim database. First, from the official health management information system, an administrative dataset managed by the Health Commission of H city, we identified all patients diagnosed with diabetes before January 2015. By linking with the patients’ identification, we extracted their social demographic information (gender, age, type of medical insurance) and the records of patients’ health care utilization and costs from the medical insurance claim database, which is managed by the Healthcare Security Bureau of H city, in the time period from 2014 to 2019.

### 2.3. Definition of complications

According to ICD-10 codes, we categorized the diagnoses into six diabetes-related disease or complication groups: (1) cerebrovascular disease, (2) cardiovascular diseases, (3) nephropathy, (4) other peripheral circulatory complications, (5) ophthalmic complications, and (6) peripheral neuropathy. If a patient had at least one claim for a complication described above in a certain year during 2014–2019, he or she was grouped as a patient with the specific complication.

### 2.4. Data analysis

#### 2.4.1. Descriptive analysis

In our study, only the direct medical costs were taken into consideration, which included the cost of diagnosis, treatment, laboratory testing, drugs (prescription or nonprescription), and medical care provided by primary health care institutions, such as hospitals, clinics, etc. The diabetes-related direct medical cost (DM cost) was defined as the cost occurring in diabetes-related medical visits. Outpatient visits, hospitalizations, and drug store consumption were categorized as “diabetes-related” visits when the diagnosis explicitly indicated diabetes, diabetic complications or diabetic related disease.

The reported costs of 2014–2019 were adjusted to 2019’s price using the Chinese official reported consumer price index [1.4% in 2015, 2.0% in 2016, 1.6% in 2017, 2.1% in 2018, and 2.9% in 2019 (20)]. All costs were reported in USD using the exchange rate of 2019 [1 USD = 6.90 Chinese Yuan in 2019 (20)]. We described DM costs and their average annual growth rates (AAGR) for patients with diabetes from 2014 to 2019, according to whether they had a hospitalization that year or whether they were classified as having any complications. Data preparation and statistical analyses were performed using Stata MP Version 17.0.

#### 2.4.2. Multiple linear regression analysis

A multiple linear regression model was employed in this study. We estimated the following equation:


$$Y_{it}=\alpha +\beta_{1}\cdot X_{i}+\beta_{2}\cdot year_{i}+\beta_{3j}\cdot Z_{ij}+\varepsilon_{it}$$

where ***Y***_***it***_ refers to the DM cost of the ***i***^***th***^ patient at year ***i***, costs were log-transformed in the model; ***X***_***i***_ refers to the key variables we attempted to explore, including hospitalization, the number of complications or the type of complications; ***year*** indicates the calendar year of the observation (2014–2019); and ***Z***_***j***_ is a vector of control variables including gender, age, types of medical insurance, whether combined with hypertension, and body mass index (BMI). The age of patients was divided into several groups: ≤30 years, 31–40 years, 41–50 years, 51–60 years, 61–70 years, 71–80 years, 81–90 years and ≥ 90 years, and the BMI states were divided according to the patients’ BMI: normal (18.5 kg/m^2^ ≤ BMI ≤ 24.0 kg/m^2^), above normal (BMI > 24.0 kg/m^2^), and below normal (BMI < 18.5 kg/m^2^). In this regression model, standard errors were clustered on the individual level.

After reversely transforming the results of the regression model, exp (***β***_***1***_) is the multiplier of a certain risk factor on the DM cost of patients with diabetes.

## 3. Results

### 3.1. Study population

Our research included 44,994 patients with diabetes (Table 1), consisting of 21,161 (47%) men and 23,833 (53%) women. The sample population was mainly composed of middle-aged and older adult, with a population aged 50–80 accounting for 85% of the cohort patients. Among the sample population, 89% of patients were covered by the UEBMI, while others were covered by the URRBMI. The number of patients with a BMI above the normal range was slightly higher than that within the normal range (49.78% vs. 48.61%), with very few people having a BMI below the normal range (1.61%).

**Table 1:** Demographic information of sampled patients in 2014.

	Sample patients
No. of patients	44,994
Gender, *N* (%)	
Male	21,161 (47.03)
Female	23,833 (52.97)
Age, Mean (SD)	65.29 (10.53)
Age group, *N* (%)	
<=30	63 (0.14)
31–40	487 (1.08)
41–50	2,830 (6.29)
51–60	11,346 (25.22)
61–70	16,066 (35.71)
71–80	10,814 (24.03)
81–90	3,290 (7.31)
> = 90	98 (0.22)
Insurance type, *N* (%)	
UEBMI	40,180 (89.30)
URRBMI	4,814 (10.70)
BMI, *N* (%)	
<18.5	726 (1.61)
18.5–24	21,870 (48.61)
>24	22,398 (49.78)

### 3.2. Prevalence of diabetic complications and related diseases

Table 2 showed the prevalence of 6 complications among patients with diabetes. Based on recorded diagnoses, the proportion of diabetic patients with complications or related diseases increased significantly, from 58.87% in 2014 to 91.90% in 2019. The proportion of patients with one complication gradually decreased, while the proportion of patients with 3 or more complications increased sharply from 2014 to 2019. In 2019, more than 40% of patients with diabetes had 2–3 complications, and more than 15% of patients with diabetes experienced 5 or more complications (almost 15 times higher than in 2014). The most prevalent complication was cardiovascular disease, followed by ophthalmic complications and peripheral circulatory complications.

**Table 2:** Prevalence of diabetic complications and related diseases (*N* = 44,994).

Percent of patients (%)	2014	2015	2016	2017	2018	2019	Increases (percentage point)
Patients with complications	58.87	72.56	80.69	85.76	89.41	91.90	33.03
With one complication	29.70	27.53	25.07	21.90	19.11	16.53	−13.17
With two complications	17.09	21.52	22.57	22.58	21.86	20.80	3.71
With three complications	8.09	13.40	16.72	18.81	20.32	20.98	12.89
With four complications	2.96	6.79	10.17	13.07	15.23	17.27	14.31
With five complications	0.89	2.69	4.68	6.89	9.15	11.33	10.44
With six complications	0.14	0.64	1.48	2.51	3.74	4.99	4.85
Prevalence of different diseases							
Cerebrovascular disease	14.05	20.96	26.48	31.49	35.70	39.54	25.49
Cardiovascular diseases	28.23	38.70	46.14	52.06	57.12	61.38	33.15
Nephropathy	14.53	20.17	25.03	28.86	32.32	35.69	21.16
Peripheral circulatory complications	19.25	28.61	36.00	42.41	47.92	52.44	33.19
Ophthalmic complications	21.05	33.49	41.91	48.52	54.07	58.29	37.24
Peripheral neuropathy	8.15	13.27	17.73	21.94	25.74	29.38	21.23

Data are presented as a percent of the total population (%).

### 3.3. Annual diabetes-related costs of patients

**Figure 1:**
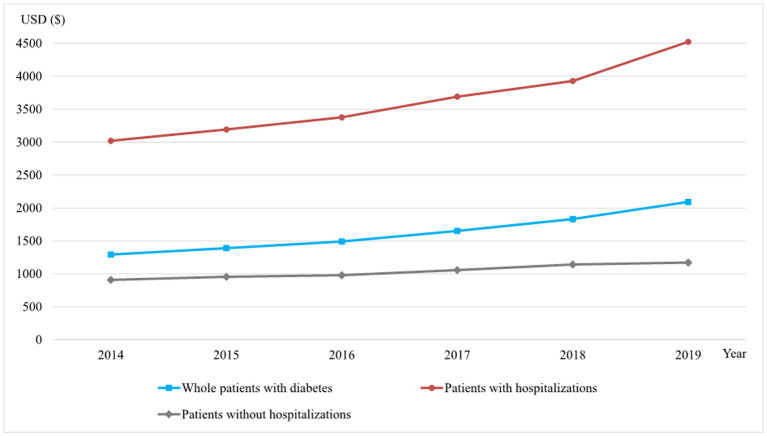
The average annual DM costs of diabetic patients with different hospitalization status, 2014–2019 (USD).

**Figure 2:**
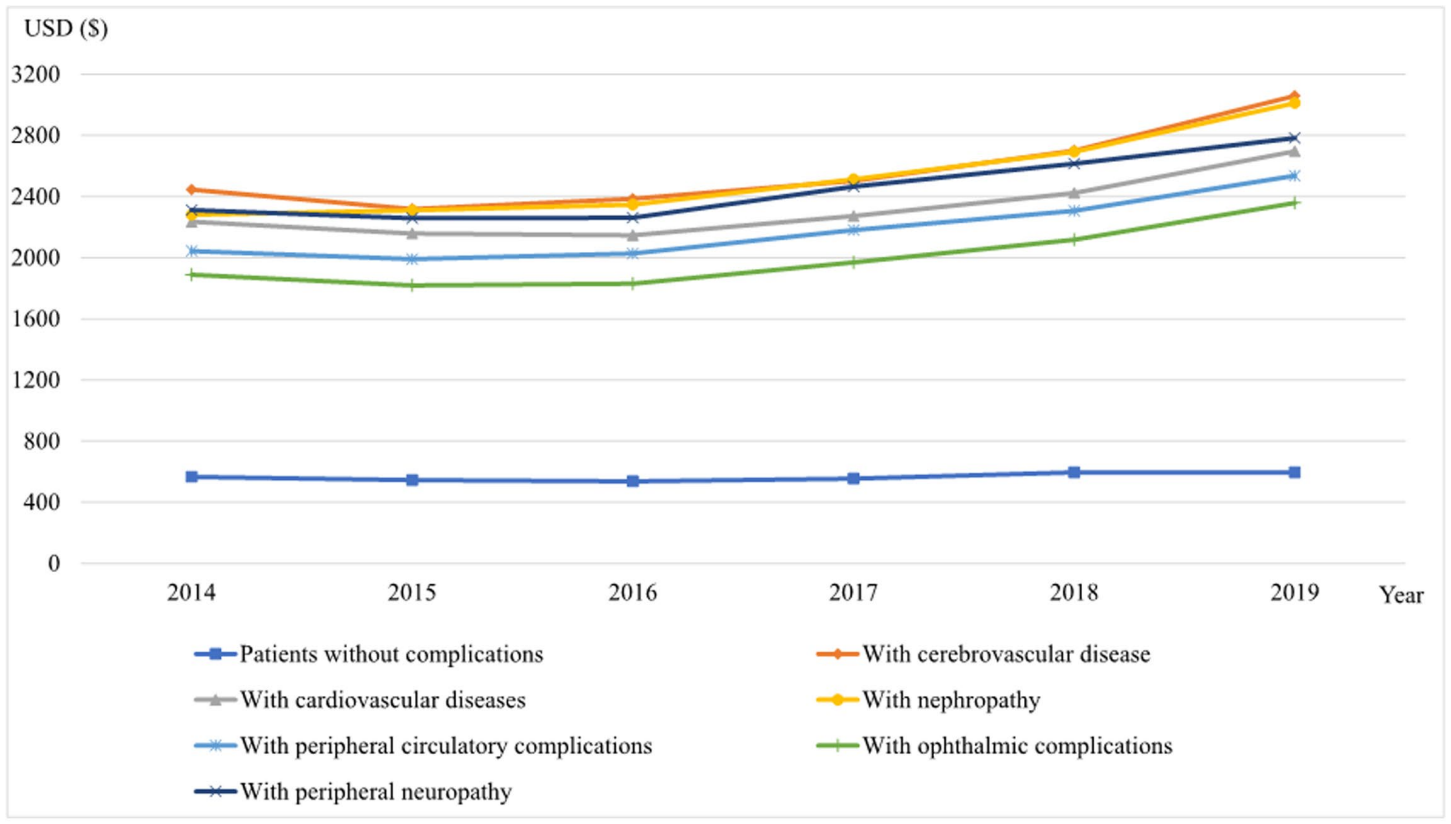
The average annual DM costs of diabetic patients with different types of diabetes-related complications, 2014–2019 (USD).

Figures 1, 2 described the trend of average annual DM costs for patients with diabetes, and the specific data could be seen in Supplementary Table S1. As shown in Figure 1, the average annual DM costs for the whole sample of diabetic patients increased from 1,292.72 USD in 2014 to 2,092.87 USD in 2019, with an average annual growth rate of 10.12%. The average annual DM cost for diabetic patients with hospitalizations was more than three times that of patients without hospitalization (4,524.27 vs. 1,171.78 USD in 2019), and both increased from 2014 to 2019, with annual increases of 8.41% and 5.24% on average, respectively.

The DM costs for patients in all complication groups showed a gradual upward trend from 2014 to 2019 (Figure 2). The DM costs of patients without complications were all lowest and almost unchanged during 2014–2019 (with an AAGR of 0.98%). Patients with cerebrovascular disease have the highest annual DM cost per patient (3,058.31 USD in 2019), followed by patients with nephropathy (3,011.55 USD in 2019), patients with peripheral neuropathy (2,783.01 USD in 2019), and patients with cardiovascular diseases (2,696.34 USD in 2019). The annual DM cost for patients with peripheral circulatory complications was 2,600.03 USD per patient, and for patients with ophthalmic complications was 2,417.52 USD per patient.

### 3.4. Factors influencing diabetes-related costs for patients with diabetes

Table 3 shows the regression coefficients of key factors in the model analysis and their impact on the DM costs of diabetic patients. The results of Model 1 and Model 2 both showed that the average annual DM cost for patients with hospitalization was 2.23 times that of patients without hospitalization. The DM cost of patients increased with the number of complications, according to the results of Model 1 (Table 3). The DM cost of patients with one complication was 1.83 times that of patients without complications, and the DM cost of patients with 2 complications was 2.70 times that of patients without complications. The DM cost of patients with 3, 4, 5, and 6 complications was 3.62 times, 4.61 times, 5.95 times, and 7.53 times more than that of those without complications, respectively. As shown by the results of Model 2 (Table 3), the impact of each complication on the patients’ DM costs was different, and all were statistically significant (*p* < 0.01). The DM costs for patients with cardiovascular complications and nephropathic complications were 1.65- and 1.54-fold higher than those of patients without the complications, respectively. Ophthalmic complications had a relatively lower impact on patients’ DM costs, increasing patient costs by 19%.

**Table 3:** Factors influencing of diabetes-related costs for patients with diabetes.

Variables	Model 1		Model 2	
	Regression coefficient (*β*)	*e^β^*	Regression coefficient (*β*)	*e^β^*
With hospitalization	0.801*** (0.007)	2.23	0.803*** (0.007)	2.23
Number of complications (contrast: without any)				
With one complication	0.605*** (0.015)	1.83		
With two complications	0.995*** (0.015)	2.70		
With three complications	1.287*** (0.016)	3.62		
With four complications	1.528*** (0.017)	4.61		
With five complications	1.783*** (0.018)	5.95		
With six complications	2.019*** (0.021)	7.53		
Type of complications				
Cerebrovascular disease (Contrast: without)			0.331*** (0.009)	1.39
Cardiovascular diseases (Contrast: without)			0.503*** (0.009)	1.65
Nephropathy (Contrast: without)			0.433*** (0.009)	1.54
Peripheral circulatory complications (Contrast: without)			0.343*** (0.009)	1.41
Ophthalmic complications (Contrast: without)			0.178*** (0.009)	1.19
Peripheral neuropathy (Contrast: without)			0.279*** (0.009)	1.32
Control variables	Yes		Yes	
Time effect	Yes		Yes	
Individual effect	Yes		Yes	
Observations (person-years)	269,964		269,964	
*R* ^2^	0.301		0.300	

The other covariates of Model 1 and Model 2 included year dummies, gender, age group dummies, types of medical insurance dummies, whether combined with hypertension, and BMI. Standard errors were clustered on the institutional level, and shown in parentheses. Significance levels: *p < 0.1; **p < 0.05; ***p < 0.01.

## 4. Discussion

Based on the official health management information system and the medical insurance claim database, our study conducted a comprehensive analysis of the economic burden of diabetes in urban areas of eastern China using longitudinal data. In this study, we used claim records to compare the 6-year economic burden and changing trends of the same cohort of patients. Our results showed an increasing trend in the DM costs for diabetic patients in urban China from 2014 to 2019. The economic burden of diabetes was significantly associated with hospitalization as well as the number and types of complications. During the period 2014–2019, the proportion of patients who required hospitalization increased steadily, and the DM costs of hospitalized patients were 2.2 times higher than those of non-hospitalized patients. In addition, the results described a striking increasing trend in the proportion of patients with complications, which increased 33 percentage points from 2014 to 2019. Furthermore, the proportion of patients with multiple complications also increased sharply—patients with three or more complications increased from 12% in 2014 to 50% in 2019. Regarding the types of complications, the proportion of patients with ophthalmic complications, cardiovascular diseases, and peripheral circulatory complications increased the most, and all of their prevalence rates exceeded 50% in 2019. Among these 6 complications, according to the regression results, diabetic patients with cardiovascular disease had an average of 65% higher DM costs than those without.

Based on stricter sample selection requirements, the distribution of characteristics of diabetic patients in our study sample is slightly different from other studies. As the purpose of this study was to track and analyze changes in health service utilization and costs in the diabetes cohort from 2014 to 2019, the patients included in the study were those who had a 6-year record of outpatient visits or hospitalizations, that is, patients receiving treatment. Some patients may have incomplete records due to discontinuation of treatment or moving in and out of H city during the study period and were not included in this study. What’s more, according to existing research, over half (53%) of adults living with diabetes are undiagnosed (2). Thus, there must be more people with diabetes in the region than in the cohort population. Therefore, we did not analyze and report the prevalence of diabetes in H city. The distribution of age and BMI in the study population was in accordance with the conclusions of the literature on the prevalence of diabetes in China (15, 17–19). In addition, compared to existing studies that investigated the prevalence of diabetic complications and related diseases in China (3), our sample showed a similar prevalence of nephropathy (35.69% in 2019), but a higher prevalence of cerebrovascular disease (39.54% in 2019), cardiovascular diseases (61.38% in 2019), peripheral circulatory complications (e.g., diabetic foot) (52.44% in 2019), ophthalmic complications (58.29% in 2019), and peripheral neuropathy (29.38% in 2019). A possible explanation for this result is that patients with multiple chronic diseases (such as hypertension or other diabetes-related diseases) are more likely to receive regular treatment for 6 consecutive years, and thus were more likely to be included in our study.

The results of this study showed that the average annual DM cost per patient increased from 1,292.72 USD in 2014 to 2,092.87 USD in 2019, which was slightly higher than the average annual cost of treating and managing diabetes per person with diabetes (20–79 years) in the western Pacific region (1,204 USD in 2021) (2). Considering that the sample city in our study is a relatively economically developed city in China, the measurement and calculation of the DM cost of diabetes in our study are credible. The average annual DM cost per patient in city H in 2019 accounted for approximately 10.98% of the GDP *per capita* of that city. The average annual growth rate of DM cost per patient was 10.12% from 2014 to 2019 in our study, which was much larger than the average annual growth rate of GDP *per capita* both in the sample city (7.19%) and in China (8.36%) during that period, which reflected the increasing economic burden of diabetes in eastern urban cities in China. Compared with other studies conducted in China, the annual direct medical costs related to diabetes per patient in our study were higher than those in Zhuhai (16), Guilin (21), and Kunming (22), and the results of a survey of 16 tertiary hospitals in 14 cities in China (23). Variations in the economic burden of diabetes patients in different regions are related to the level of economic development, medical resource allocation, chronic disease prevention and management policies in different regions, and the different data sources and investigation years in different studies. The above comparison can also reflect the high consumption of medical resources by patients with diabetes in the relatively developed cities along the eastern coast of China.

The results of our study confirmed that hospitalization and complications play a decisive role in the diabetes-related direct medical costs of diabetic patients, which is consistent with previous studies (15, 23–28). Hospitalization is essentially a reflection of the severity of a diabetic patient’s condition and a manifestation of poor control and management of diabetes (29). The findings revealed that the diabetes-related direct medical costs of patients who were hospitalized were higher than those of patients who were not, indicating a greater financial burden for them. Our study also described an increasing trend in the number of patients needing hospitalization during 2014–2019, which highlights the poor status of diabetes prevention and the need for further strengthening local chronic disease management. In terms of complications, our research shows that the number and types of complications in patients with diabetes have a direct impact on their diabetes-related direct medical costs. This result highlights evidence from other studies that the medical costs caused by complications contribute to a high economic burden for patients with diabetes (15, 23–28). The results of this study showed that the proportion of both patients with at least one complication and patients with multiple complications increased significantly from 2014 to 2019. It has been demonstrated that diabetic patients with multiple complications have a significantly increased risk of hospitalization and premature death, and their financial burden is much higher than that of patients without complications (10, 15, 28). Our results confirm that minimizing diabetes complications can have substantial financial compensation.

Compared with existing studies, our study has several obvious advantages. Firstly, our research enriches the findings on the economic burden in an eastern city in China, and provides a more comprehensive understanding of the trends in the economic burden of diabetes over time, compared to cross-sectional studies that only examine the burden at a single point in time. Secondly, the large sample size increases the generalizability of the findings and reduces the risk of random error in the results. Thirdly, this study explores the influence of hospitalization and the number and types of complications on the DM cost of diabetes by constructing a multiple regression model and controlling for other factors that may have an impact on patients’ costs. Finally, the electronic claim database we used includes information on patients’ visits to health institutions at all levels, which can provide a more accurate picture of the overall economic burden of the disease, which is the main limitation of studies based on individual hospitals.

However, this study still has limitations. Firstly, there may be selection bias in the sample if only individuals with health insurance or those who seek medical treatment are included in the study. This can result in an underestimation of the overall number of diabetics in the area. Secondly, as only one city in the developed region of China was selected as the sample in our study, the findings may only be generalizable to the specific population and healthcare system in China, and may not be directly applicable to other countries or regions. Thirdly, we did not examine the indirect costs associated with diabetes patients since our claim database is unsuitable for computing them. The changes in the indirect cost of diabetes and its influencing factors will be an important aspect of future research.

## 5. Conclusion

The economic burden of diabetes in urban China has increased significantly, and the hospitalization rate and the prevalence of diabetes complications, which have direct impacts on the cost of diabetes, also present a constantly increasing trend. To reduce the growing economic burden of diabetes, efforts should be invested in screening mechanisms and patient management programs to prevent long-term complications in patients with diabetes.

## Data availability statement

The datasets presented in this article are not readily available because of the non-disclosure agreements which provided the data. Requests to access the datasets should be directed to WC, wenchen@fudan.edu.cn.

## Ethics statement

The studies involving human participants were reviewed and approved by The Ethics Review Committee of the School of Public Health, Fudan University approved our study (IRB#2016-03-19). Since the data we used came from two anonymized and secondary databases, human participants were not directly involved in the study, and the informed consent was exempted. Written informed consent for participation was not required for this study in accordance with the national legislation and the institutional requirements.

## Author contributions

WC and LZ contributed to the conception and design of the manuscript and data collection. XL cleaned and analyzed the data. XL and LZ wrote the first draft. WC and LZ reviewed and edited the manuscript. All authors contributed to the article and approved the submitted version.

## Funding

This research was funded by “The National Social Science Foundation of China, grant number 20ZDA072, “The Shanghai Philosophy and Social Science Research Program, grant number 2020BGL003” and “The Shanghai Pujiang Program, grant number 21PJC024.”

## Conflict of interest

The authors declare that the research was conducted in the absence of any commercial or financial relationships that could be construed as a potential conflict of interest.

## Publisher’s note

All claims expressed in this article are solely those of the authors and do not necessarily represent those of their affiliated organizations, or those of the publisher, the editors and the reviewers. Any product that may be evaluated in this article, or claim that may be made by its manufacturer, is not guaranteed or endorsed by the publisher.

## Abbreviations

AAGR: Average annual growth rate
BMI: Body mass index
DM cost: Diabetes-related direct medical cost
GDP: Gross Domestic Product
UEBMI: Urban Employee Basic Medical Insurance
URRBMI: Urban and Rural Residents Basic Medical Insurance
USD: US dollars
