# Supplementary material for: Trends in economic burden of type 2 diabetes in China: Based on longitudinal claim data
Source: Front Public Health. 2023 Apr 18;11:1062903. doi: 10.3389/fpubh.2023.1062903 (PMC10151735; doi:10.3389/fpubh.2023.1062903)
Supplement: Supplementary file 1 [file Table_1.DOCX]

Supplementary Material

**Supplemental table S1. The average annual DM costs for diabetic patients in stratified patient groups during 2014-2019 (USD)**

| **Items** | **2014** | **2015** | **2016** | **2017** | **2018** | **2019** | **AAGR** |
| --- | --- | --- | --- | --- | --- | --- | --- |
| **Annual costs per patient** |  |  |  |  |  |  |  |
| Number of patients, N | 44994 | 44994 | 44994 | 44994 | 44994 | 44994 |  |
| DM cost, Mean (SD) | 1292.72 (2460.43) | 1390.96 (2103.83) | 1492.00 (2631.30) | 1652.01 (2766.10) | 1831.87 (3225.50) | 2092.87 (4197.83) | 10.12% |
| **Annual costs per patient of patients with or without hospitalizations** | | |  |  |  |  |  |
| **Patients with hospitalizations** |  |  |  |  |  |  |  |
| Number of patients, N | 8197 | 8768 | 9614 | 10174 | 11142 | 12362 |  |
| DM cost, Mean (SD) | 3020.94 (3874.87) | 3192.33 (3893.67) | 3377.77 (4976.53) | 3690.09 (5012.23) | 3928.96 (5719.87) | 4524.27 (7278.50) | 8.41% |
| **Patients without hospitalizations** |  |  |  |  |  |  |  |
| Number of patients, N | 36797 | 36226 | 35380 | 34820 | 33852 | 32632 |  |
| DM cost, Mean (SD) | 907.73 (1801.21) | 954.96 (923.47) | 979.57 (920.34) | 1056.51 (989.28) | 1141.63 (1066.08) | 1171.78 (1068.40) | 5.24% |
| **Annual DM costs per patient of patients with each complication, Mean (SD)** | | | |  |  |  |  |
| Patients without complications | 566.24 (615.82) | 544.51 (524.08) | 536.53 (553.69) | 554.18 (599.32) | 595.19 (797.13) | 594.66 (643.63) | 0.98% |
| With cerebrovascular disease | 2444.81 (3074.77) | 2317.95 (2828.18) | 2385.74 (3858.78) | 2502.48 (3699.16) | 2700.95 (4239.70) | 3058.31 (5532.66) | 4.58% |
| With cardiovascular diseases | 2235.67 (2919.66) | 2157.54 (2819.22) | 2146.60 (3302.16) | 2272.49 (3392.02) | 2422.04 (3824.88) | 2696.34 (4861.35) | 3.82% |
| With nephropathy | 2280.86 (2676.93) | 2309.07 (2862.66) | 2346.90 (3697.20) | 2513.94 (3660.69) | 2692.16 (3987.14) | 3011.55 (5105.03) | 5.72% |
| With peripheral circulatory complications | 2043.37 (2629.23) | 1990.38 (2615.19) | 2026.69 (3323.53) | 2179.87 (3328.40) | 2306.32 (3614.03) | 2536.28 (4504.76) | 4.42% |
| With ophthalmic complications | 1888.11 (3904.02) | 1818.42 (2320.04) | 1829.83 (2972.70) | 1968.62 (2912.89) | 2118.36 (3374.16) | 2358.25 (4324.89) | 4.55% |
| With peripheral neuropathy | 2311.60 (2594.47) | 2258.37 (2572.18) | 2261.33 (2954.92) | 2465.43 (3405.25) | 2614.55 (3797.32) | 2783.01 (4629.56) | 3.78% |

Note: DM costs: diabetes-related costs, AAGR: Average annual growth rate
